# Stress Adaptive Plasticity: *Aegilops tauschii* and *Triticum dicoccoides* as Potential Donors of Drought Associated Morpho-Physiological Traits in Wheat

**DOI:** 10.3389/fpls.2019.00211

**Published:** 2019-02-25

**Authors:** Yadhu Suneja, Anil Kumar Gupta, Navtej Singh Bains

**Affiliations:** ^1^Department of Biochemistry, Punjab Agricultural University, Ludhiana, India; ^2^Department of Plant Breeding and Genetics, Punjab Agricultural University, Ludhiana, India

**Keywords:** *Aegilops tauschii*, *Triticum dicoccoides*, water stress, genetic variation, stress adaptive plasticity, root-shoot development, proline induction, membrane injury

## Abstract

The inconsistent prevalence of abiotic stress in most of the agroecosystems can be addressed through deployment of plant material with stress adaptive plasticity. The present study explores water stress induced plasticity for early root-shoot development, proline induction and cell membrane injury in 57 accessions of *Aegilops tauschii* (DD-genome) and 26 accessions of *Triticum dicoccoides* (AABB-genome) along with durum and bread wheat cultivars. Thirty three *Ae. tauschii* accessions and 18 *T. dicoccoides* accessions showed an increase in root dry weight (ranging from 1.8 to 294.75%) under water stress. Shoot parameters- length and biomass, by and large were suppressed by water stress, but genotypes with stress adaptive plasticity leading to improvement of shoot traits (e.g., *Ae tauschii* accession 14191 and *T. dicoccoides* accession 7130) could be identified. Water stress induced active responses, rather than passive repartitioning of biomass was indicated by better shoot growth in seedlings of genotypes with enhanced root growth under stress. Membrane injury seemed to work as a trigger to activate water stress adaptive cellular machinery and was found positively correlated with several root-shoot based adaptive responses in seedlings. Stress induced proline accumulation in leaf tissue showed marked inter- and intra-specific genetic variation but hardly any association with stress adaptive plasticity. Genotypic variation for early stage plasticity traits viz., change in root dry weight, shoot length, shoot fresh weight, shoot dry weight and membrane injury positively correlated with grain weight based stress tolerance index (*r* = 0.267, *r* = 0.404, *r* = 0.299, *r* = 0.526, and *r* = 0.359, respectively). In another such trend, adaptive seedling plasticity correlated positively with resistance to early flowering under stress (*r* = 0.372 with membrane injury, *r* = 0.286 with change in root length, *r* = 0.352 with change in shoot length, *r* = 0.268 with change in shoot dry weight). Overall, *Ae. tauschii* accessions 9816, 14109, 14128, and *T. dicoccoides* accessions 5259 and 7130 were identified as potential donors of stress adaptive plasticity. The prospect of the study for molecular marker tagging, cloning of plasticity genes and creation of elite synthetic hexaploid donors is discussed.

## Introduction

Alternate morpho-physiological manifestations of genes in response to specific environmental cues represent a key adaptation and survival strategy among plants, offsetting their limited capability to change the growth environment. This strategic property often referred to as plasticity has been defined as the ability of a single genotype to produce more than one phenotype in response to environment (Bradshaw, 1965). Plant phenotypic plasticity can be either a passive consequence of resource availability, physical conditions etc, or an active (adaptive) response to these conditions (Des Marais and Juenger, 2010). The latter generally implies specific development, physiological and reproductive adjustments that are thought to optimize plant fitness (Pacheco-Villalobos and Hardtke, 2012). Extensive above ground architectural changes in response to biotic and abiotic factors (Tomlinson and O’Connor, 2004), shifts in patterns of root development in search of nutrients and moisture (Lloret et al., 1999; Hodge, 2004), exudation of metabolites by roots for nutrient acquisition (Metlen et al., 2009), accumulation of osmolytes (Seki et al., 2007), modulation of stomatal density (Hetherington and Woodward, 2003), changes in leaf pigmentation (Chalker-Scott, 1999) are some of the well recognized examples of adaptive plasticity in plants. In study of natural plant populations, phenotypic plasticity is no longer seen as a source of noise (Nicotra and Davidson, 2010) in fact, it is receiving increasing recognition as a feature of ecological and evolutionary significance (de Jong, 2005; Bradshaw, 2006; Lande, 2009; Nicotra et al., 2010; Des Marais et al., 2013; Aspinwall et al., 2014; Bloomfield et al., 2014; Matesanz and Milla, 2018).

Genetic variation in plasticity in response to abiotic stress, particularly in model plant species like *Arabidopsis thaliana* (Mouchel et al., 2004; Kesari et al., 2012) and *Brachyipodium distachyon* (Luo et al., 2011; Pacheco-Villalobos and Hardtke, 2012) is well indicated. Genetic variation has been identified in natural accessions of Arabidopsis for *Bravix Radix (BRX)* locus, a transcription factor responsible for controlling root proliferation and its elongation (Mouchel et al., 2004; Beuchat et al., 2010). Using Recombinant inbred lines (RILs), two robust QTLs, *EDG1*, and *EDG2* (elicitors of drought growth) contributing to plasticity in root system size under mild osmotic stress were identified in Arabidopsis (Fitz Gerald et al., 2006). Also, Pajoro et al. (2017) envisaged the contribution of transposons and alternative splicing toward thermoplasticity in flower development in natural accessions of Arabidopsis. Crop scientists are just beginning to embrace the plasticity concept (Sardas et al., 2009; Melino et al., 2015). With respect to plasticity in crop species Nicotra et al. (2010) have raised two outstanding questions. First seeks to understand if crop breeding has led to reduction in adaptive plasticity. When the impact of breeding on phenotypic plasticity of oat’s varieties was examined, modern varieties (as compared to the older ones) showed least plasticity in stem elongation in response to variation in light conditions (Semchenko and Zobel, 2005). Information so far is, however, insufficient for a consensus to be reached on this issue. The second question seeks to know if we can breed crops for plasticity in key traits with the ultimate goal of improving yield stability under climate perturbations. Key functional traits such as leaf mass per unit area, stomatal size and density, plant height at maturity, flowering time, seed size, water use efficiency, leaf morphology, root to shoot ratio, plant chemical defenses etc. have been recommended for investigation of adaptive phenotypic plasticity. Several studies are now addressing these and other related questions. Ehdaie et al. (2012) for instance, evaluated a set of near isogenic wheat-rye translocation lines for root allocation and plasticity under well watered and drought conditions and found adaptive phenotypic plasticity of root system components to reduce negative impact of drought stress on grain yield. Integrating these researches into practical cereal breeding is likely to emerge as a major future requirement.

Wheat, as a crop, epitomizes the effectiveness of the genetic strategy in food-securing the world in the face of increasing population and rising per capita consumption. In wheat, as in other green revolution crops, enhanced productivity was largely achieved through selection for performance in a specific environmental situation represented by assured and high input use. This strategy, however, proved less effective for the inherently variable drought stress environments which represent a substantial proportion of wheat growing regions of the world (Ludlow and Muchow, 1990). Presently, besides tolerance to natural stress, there is also a need to develop genotypes adapted to low input use in the view of environmental and resource depletion concerns. Trait plasticity may prove useful to buffer productivity in the face of unmanageable spatial and temporal variations in production conditions. Trait plasticity is thus an attractive prospect in the light of sustainable agriculture but donor options in the cultivated germplasm are likely to be constrained owing to the selection regimes historically employed. In case of wheat, severe genetic bottlenecks were imposed by rare interspecific hybridization and polyploidization events accompanying bread wheat domestication (Cox, 1997). As a result, lower levels of polymorphism are observed for many traits in common wheat in comparison with its progenitor species (Kam-Morgan et al., 1989). The three wheat genomes (A-, B-, and D-) of cultivated wheat have their ancestral complements enshrined in two immediate progenitor species, *Aegilops tauschii* (DD-genome donor) and *T. dicoccoides* (AABB-genome donor). Since the potential for recombination based gene transfers from progenitor species is enormous (as compared to non-progenitor donors), incorporation of adaptive plasticity traits from these wheat progenitors could greatly expand the available domesticated gene pool and enrich the possibilities of combining resilience and productivity of wheat varieties making them perform better over a range of predictable and unpredictable environment regimes.

With these points in mind, the primary objective of the present study was to identify genetic variation for “water stress adaptive plasticity” in a set of accessions belonging to two species which are the immediate wild progenitors of hexaploid wheat. Productivity/yield oriented indices generally employed as a measure of stress adaptation in cultivated wheats would not be relevant for this set. Considering the nature of target traits as well as the plant material, lab based assays were seen to be more appropriate. Accordingly seedling traits (length and biomass of both root and shoot) formed the core of the experiment for studying stress induced plasticity. In a second experiment, two characters based on leaf tissue (proline content and cell membrane injury) were assayed at vegetative stage from field grown plants. Leaf tissue could be conveniently sampled, irrespective of species differences and field stress provided the required induction of tolerance mechanisms. A third experiment dealt with field observations on flowering time, plant growth (height) and yield components (spike length and grain weight). This experiment was aimed at relating stress adaptive changes in seedling and early/vegetative stage with one or more productivity based indices of stress tolerance. In all the experiments, “change in trait value” across well watered and water stress conditions rather than the absolute values formed the basis of analysis. This helped us to focus on “stress adaptive plasticity” and also make comparisons across species. Further, as the absolute values of these parameters vary greatly across the three species employed here, comparable observations were generated in the form of “change in trait value” across well watered (WW) and water stress (WS) conditions. The study reports wide variation both within and between the species, trait interactions and trade-offs, demarcation of potential donors for use in breeding program and considerations for a wheat improvement strategy.

## Materials and Methods

### Choice of Germplasm

The wild progenitor species germplasm used in the present study consisted of 57 accessions of *Aegilops tauschii* and 26 accessions of *Triticum dicoccoides*. The two germplasm sets are listed in Supplementary Tables S1a,b with respect to their pau gene bank accession numbers. To refer to a particular accession in the text, numeral component of the designation is used. *Aegilops tauschii* and *Triticum dicoccoides* germplasm maintained at Punjab Agricultural University was received from different sources (University of Missouri and Kansas State University, United States; IPK, Gatersleben, Germany; Centre for Plant Breeding Research, Wageningen, Netherlands and National Bureau of Plant Genetic Resources, New Delhi, India) over a period of time (Supplementary Tables S1a,b). Subsets of this material have been subjected to screening for genetic variation for acquired thermotolerance with respect to cell membrane stability and TTC based cell viability (Gupta et al., 2010), disease resistance and high molecular weight glutenin subunits (Chhuneja et al., 2010), alleles of vernalization genes at *VRN-A1* and *VRN-B1* loci (Chhuneja et al., 2015) and detection of SNPs for grain size variation (Arora et al., 2017) in earlier studies at our center.

Cultivars of bread wheat- PBW-343, PBW-550, PBW-621, C-306, and durum wheat- PDW-291, PDW-314, and WHD-943 were included in present study as reference material (Supplementary Table S1c), with which attributes of wild accessions were compared. As the number of cultivated lines was considerably smaller, their comparison with the progenitor sets (in relation to spectrum of variation) may not be fully justified though some reprieve was provided by the deliberate inclusion of tall, traditional rain-fed cultivar (C-306) along with modern day high productivity varieties (PBW-343, PBW-550, and PBW-621) recommended for cultivation under irrigated conditions. Similarly, inclusion of durums (PDW-291, PDW-314, and WHD-943) along with bread wheat cultivars added an element of variation which might have taken a much larger random set of cultivated wheats to encompass.

### Evaluation of Stress Adaptive Plasticity of Different Accessions

#### Seedling Assays

For evaluating plasticity in root-shoot development under water stress conditions, a preliminary experiment was carried out to optimize the concentration (10, 15, 20, and 25%) of polyethylene glycol (PEG) solution for the current study. A parallel set up involving different concentrations of mannitol was also used (2, 3, 4, 5, and 6%). Twenty five per cent PEG was found to cause about 50% reduction in growth and thereafter, this concentration was used for the complete study. Responses of 14 day old seedlings were observed in terms of length and weight of both roots and shoots under well watered and water stress conditions. Before deciding on the use of propagation trays, a subset of 23 accessions (twelve *Ae. tauschii*, seven *T. dicoccoides*, two bread wheat and two durum wheat cultivars) were grown in three types of containers, namely, propagation trays, small cups and root trainer trays. The genotypic values for root and shoot growth under both well watered and water stress conditions correlated well across container systems (Supplementary Table S2). As the growth studies in propagation tray involved shorter time frame and required lesser space, further study on complete set was carried out in this mode. This set up would favor use of seedling assay, if need be, on a breeding scale.

For the present study, seven seeds (each) of *Ae. tauschii* and *T. dicoccoides* accessions along with check wheat cultivars were sown in triplicate (a total of 21 seeds per accession in each water regime) in two sets of propagation trays (with adequate size to support root-shoot growth for about 2 weeks) where one set served as control (well-watered) and the other set was subjected to polyethylene glycol (PEG) based water stress. Three replicates (where seven seedlings constituted one biological replicate) were sown in two sets of propagation trays in completely randomized design (CRD). These trays were placed in a Conviron growth chamber PGR15 maintained at a temperature of 25 ± 2°C and a light intensity of 400 μmol m^-2^ s^-1^. Both the sets were watered with one-fourth strength Murashige and Skoog (MS) salt solution for first week (normal irrigation). From 8^th^ day onwards (when seedlings were 5–7 cm long), 25% poly ethylene glycol (PEG-6000) in 0.25 × MS solution was used as the moisture stress inducing medium (-0.95 MPa) (modified from Blum et al., 1980). Thereafter, every alternate day, two sets were watered with 0.25 × MS medium (well watered) and 25% PEG solution in 0.25 × MS medium (water stress), respectively, till next 7 days when the tissue was harvested and the data was eventually recorded for root length, root biomass (fresh and dry weight), shoot length and shoot biomass (fresh and dry weight) under both well watered and water stress conditions. One of the *T. dicoccoides* accessions 14004-2 (Supplementary Table S1b), however, did not germinate and was excluded from the seedling based assay.

#### Field Study

With regard to field based investigation, 30 seeds of *Ae. tauschii* accessions were initially sown in propagation trays in first week of September (10-09-2010 and 08-09-2011) and kept for vernalization in cold chambers maintained at 4°C with 8/16 h light/dark regime for 7 weeks (Suneja et al., 2015b, 2017). After vernalization treatment, plants were conditioned at 15°C and 8/16 h light /dark for 1 week. Vernalized seedlings of *Ae. tauschii* and germinated seedlings of *T. dicoccoides* were then transplanted in the experimental fields of Department of Plant Breeding and Genetics, P.A.U. Ludhiana (30°54′N and 75°48′E) in two sets (later demarcated as irrigated/well watered and rain-fed/water stress) in the first week of November (05-11-2010 and 03-11-2011). The soil type of the experimental area is sandy loam and soil is non-saline with slightly alkaline pH of 8.0 and organic carbon content of 0.4%.

Two ridges with 3 m wide buffer zone were maintained between different irrigation treatments. Further, non-experiment border rows were planted (at margins) to take care of any seepage effect that might have arisen. A total of five plants per accession (in each replicate) were transplanted to constitute one plot. Therefore, a total of 30 germinated seedlings of each accession were transplanted (15 irrigated and 15 rain-fed) in randomized complete block design (RBD). After 45 days of transplanting, natural day length was supplemented with arrangement of halogen incandescent lamps and fluoroscent lights in field at regular spacing. Lights were switched on prior to sunset till late night to provide about 16 h of continuous light per day. This extension of light hours during short day winters of North India allowed wild species to flower normally. Cultivated wheat lines were incorporated in this set through seeding as practiced conventionally. Unlike the wild wheat progenitors, particularly *Aegilops tauschii*, that are typically adapted to temperate environment and have “winter” growth habit, cultivated wheats grown in tropical and sub-tropical regions of India are “spring” wheats. Therefore, wild and cultivated spring type accessions were handled differently with respect to crop raising practice.

Standard agronomic practices as followed for irrigated wheat in the region formed the basis of irrigation to non-stressed plots throughout the crop season. Briefly, after one round of heavy, pre-sowing irrigation (10 cm), four more rounds of irrigation (7.5 cm each) were given to the crop at 4–5 weeks interval depending upon the rainfall. The water stressed rain-fed set, on the other hand, received water only from rain as all the irrigation (except pre-sowing irrigation) was completely withheld throughout the season. During each crop season, the per cent moisture content in soil was determined gravimetrically at maximum tillering stage (about 60 days after sowing) from 6 different field locations (one from each replicate in each treatment) at four different depths- 0–30 cm, 30–60 cm, 60–90 cm, and 90–120 cm (Supplementary Table S3). At maximum tillering stage, a 36.7% difference in soil moisture content between well watered and water stress replicates during 2010–11 and a 66.4% difference in soil moisture content in 2011–12 was recorded.

**FIGURE 1:**
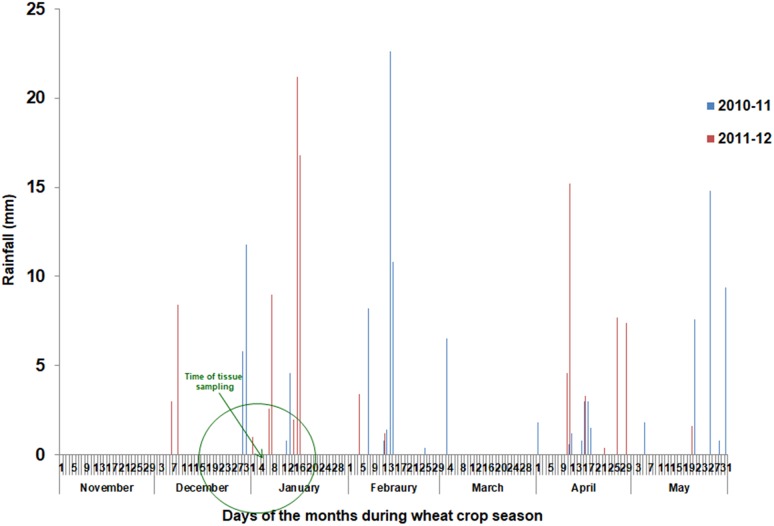
Rainfall pattern during period of field experimentation.

Overall, the crop season 2010–11 received a total rainfall of 128.8 mm, while 108.8 mm rainfall was recorded in 2011–12. Month-wise distribution of rainfall during two crop seasons has been indicated in Figure 1. For two leaf tissue based traits which were sampled at Zadok GS30 stage (about 60 days after transplanting), there were two rainfall episodes just ahead of sampling during 2010–11 while in 2011–12, almost a month long rain-free period was available prior to sampling, as seen in Figure 1. For these traits, observations from 2011 to 2012 were used for exploring genotypic variation for stress adaptive plasticity. For all other field based traits, observations from both years were used for analysis. The crop was harvested in the first week of May during both the years. As maturity in wild accessions is staggered and that seeds shatter on maturity, net bags were put on spikes 20 days prior to harvesting.

(i) Estimation of proline
  For determination of proline content, five fully expanded penultimate leaves (second leaf from the top) were collected from field during vegetative stage (60 days after transplanting) corresponding to Zadoks growth stage GS30 (Zadoks et al., 1974). Briefly, 100 mg of the leaf tissue was weighed, homogenized in 3% aqueous sulfosalicyclic acid and the content of proline was estimated using Ninhydrin reagent assay (Bates et al., 1973). Leaf proline content was estimated under well watered and water stress conditions and degree of proline induction under water stress was calculated to provide a measure of metabolic plasticity.
(ii) Estimation of Membrane Injury
  The assay for percent membrane damage was performed as mentioned in Suneja et al. (2017). For this, four fully expanded penultimate leaves (5 cm long) per accession (randomly from five plants of each plot) from rain-fed replications (Zadok growth GS30 corresponding to 60 days after transplanting) were distributed into two sets, i.e., three replicates each of control (deionised water) and *in vitro* stress treatment (40% PEG). After 24 h of PEG treatment, conductivity (μ siemens) was recorded, respectively, for control and stressed samples using a digital conductivity meter. Membrane injury index, as an indicator of cell membrane instability was worked out and membrane damage was expressed in per cent units as:

$$\text{Membrane injury index}=1-\frac{1-\left(\frac{\text{T}1}{\text{T}2}\right)}{1-\left(\frac{\text{C}1}{\text{C}2}\right)}\times 100$$

T_1_, T_2_ = Mean conductivity of stressed sample before and after autoclaving, respectively.

C_1_, C_2_ = Mean conductivity of control sample before and after autoclaving, respectively.

Hence, stress adaptive plasticity measures of all accessions belonging to wild and cultivated wheat species were estimated on the basis of observations recorded under well watered and water stress conditions (both in case of seedling based lab assays and field based screening of accessions). The estimate of stress adaptive plasticity was obtained by expressing the trait value under stress as percentage of non-stress value for the same accession. No change due to water stress was given a value of 0. Reduction in trait value under stress would lead to a plasticity measure of less than 0 (negative value). A truly stress adaptive response, say in case of root growth would be indicated by an increase in root size under water stress and consequently a plasticity value of greater than 0 (positive change) was assigned to it. The criteria thus used, favored stress adaptive changes to be elucidated rather than changes irrespective of direction. Further, using non-stress (for well-watered conditions) trait value from the same accession as a benchmark and employing change in trait value rather than absolute value as a measure for analysis made comparison across species (which vary in size and morphology) possible.

### Observations on Productivity Associated Traits and Derivation of Water Stress Tolerance Indices

Observations were recorded for days to flowering, plant height and length of the spike in field grown crop under both well watered and water stress conditions. Days to flowering was recorded as the number of days taken for 50% ear emergence from the date of transplanting. After the completion of flowering, plant height (in cm) was measured from base of the plant to the tip of the spike excluding awns and recorded as average height of five plants per accession for each replication. Spike length (in cm) was measured from neck node to the tip of the uppermost spikelet excluding awns and recorded for five spikes of each accession for each replication. For measurement of grain weight, the seeds were dehulled from spikelets of *Ae.tauschii* and *T. dicoccoides* and threshed out from ears of cultivated wheats to obtain 100-grain weight. Morphological tolerance indices were calculated on the basis of change in days to flowering (Tolerance Index 1), change in spike length (Tolerance index 2), change in plant height (Tolerance index 3) and change in grain weight (Tolerance index 4) from trait values under well watered and water stress conditions.

### Statistical Analysis

Analysis of variance (ANOVA) was performed to determine the role of genotypes (G), water stress regimes (W) and G x W interactions on variability in the expression of different drought-adaptive morpho-physiological traits (*P* ≤ 0.05, DSASTAT software 1.101). With respect to responsiveness under stress, boxplots and frequency distribution histograms were developed (using R statistical package and MS Excel 2007) for each trait to determine the extent of natural genetic variation both within and between the three groups of germplasm- *Ae. tauschii*, *T. dicoccoides* and check wheat cultivars. Using R version 3.5.1, best linear unbiased predictors (BLUPs) were obtained. When estimating BLUPs using random effect “ranef” command in lme4 package in R, variance components for all traits were analyzed using general linear mixed model to determine the effect of genotype, year and genotype × year interaction separately for well watered and water stress conditions.

The estimated phenotypic BLUP values were further used to perform correlation analysis and hierarchical cluster analysis (HCA). Correlation coefficients for the complete set (excluding three genotypes as data for a few traits was missing) were computed for associating tolerance behavior with early stage adaptive plasticity (change in trait expression) under water stress using SPSS 16.0 (IBM Corp., Armonk, NY, United States). For multivariate analysis and for overall assessment of accession-specific response to each water stress adaptive trait, a heat map was generated (using JMP14) that allowed hierarchical clustering (following Ward’s Method) of all the wild accessions and check wheat cultivars.

## Results

The germplasm set consisting of diploid (*Ae. tauschii*) and tetraploid (*T. dicoccoides*) progenitor species along with check wheat cultivars was subjected to evaluation of plastic responses in relation to water stress. A laboratory based experiment was aimed at recording adaptive changes in root and shoot growth (length and biomass) in response to stress. A second set of observations evaluated membrane injury and degree of proline induction at vegetative stage (about 60 days after transplanting) under field conditions. A third set of observations were recorded on flowering time, plant height, spike length and grain weight under irrigated and rain-fed conditions to assess variation in genotypic responses measured as difference in trait value under stress and non-stress conditions. While the first two sets of traits aimed at uncovering active adaptive responses, the third including time to flower, plant height and yield components represented impact of these responses in terms of tolerance to stress. The overall aim was to establish genetic variation for stress adaptive plasticity using low environmental noise, easy to observe traits. A further premise was that this early stage adaptive plasticity might reflect in the performance based tolerance indices.

As per the analysis of variance (Supplementary Table S4), the genotypes (G) constituting the germplasm set varied significantly for all seedling based growth traits. Trait expression for length and biomass attributes was significantly affected by water stress regimes (W) reflecting sufficient contrast maintained across the two treatments. A significant G X W interaction was observed for all evaluated traits indicating differential response of genotypes to the two water regimes. Tables 1, 2 summarizes information on mean values and range of response of the two wild species along with check wheat cultivars for lab based seedling growth assays and field based physio-biochemical evaluations, respectively.

**Table 1:** Genotypic variation in wheat germplasm set for lab based seedling growth traits recorded under well watered and water stress conditions induced by 25% PEG treatment.

Traits	Water stress regimes	*Aegilops tauschii*		*Triticum dicoccoides*		Check wheat cultivars		Full set	
		Mean	Range	Mean	Range	Mean	Range	Grand mean	LSD (0.05)
Root length (cm)	WW	3.82	1.13–6.20	5.40	2.23–8.02	5.66	4.97–7.28	4.41	1.12
	WS	3.66	1.74–6.17	5.26	2.23–7.03	7.27	5.34–9.63	4.40	1.05
Root fresh weight (mg)	WW	329.81	50.00–890.00	221.45	55.00–707.00	1944.76	1110.00–2586.67	426.39	186.23
	WS	296.03	128.00–533.33	272.97	49.00–840.60	1157.62	731.67–1493.33	357.32	163.74
Root dry weight (mg)	WW	47.77	19.05–112.50	42.44	15.00–109.00	149.76	98.33–221.67	54.30	31.86
	WS	56.59	30.00–122.50	56.49	22.33–103.25	135.00	113.33–151.67	62.73	30.41
Shoot length (cm)	WW	19.59	9.90–27.72	19.47	12.35–23.57	15.04	10.92–22.97	19.20	3.48
	WS	11.12	7.17–16.75	13.90	9.23–16.43	12.38	10.18–15.37	12.00	1.54
Shoot fresh weight (mg)	WW	1828.27	460.00–3790.00	1307.31	573.33–2343.07	2482.62	1476.67–3110.00	1733.39	345.80
	WS	666.24	235.00–1225.00	830.46	480.00–1836.67	766.19	585.00–1043.33	720.23	169.37
Shoot dry weight (mg)	WW	274.10	80.00–920.00	143.21	50.00–287.29	279.52	215.00–355.00	237.76	85.90
	WS	185.35	70.00–370.00	144.58	33.33–360.00	175.24	148.33–211.67	173.10	54.48

*WW, well watered; WS, water stress.*

**Table 2:** Genotypic variation in wheat germplasm set for leaf tissue based traits (stress developed under field conditions) recorded under well waterd and water stress conditions.

Traits	Water stress regimes	*Aegilops tauschii*		*Triticum dicoccoides*		Check wheat cultivars		Full set		
		Mean	Range	Mean	Range	Mean	Range	Grand mean	LSD (0.05)	h^2^
Proline content (mg g^-1^ FW)	WW	0.52	0.12–1.01	0.13	0.04–0.68	0.11	0.06–0.16	0.38	0.38	0.69


	WS	1.12	0.25–2.53	0.33	0.16–0.53	0.44	0.40–0.56	0.85	0.85	0.56
Membrane Injury (%)	*In vitro* stress	51.74	21.43–66.86	73.00	43.46–87.19	76.25	58.27–89.38	59.94	14.62	0.72

*h^2^, Repeatability calculated over two crop seasons 2010–11 and 2011–12. WW, well watered; WS, water stress.*

### Stress Adaptive Plasticity in Seedling and Tissue Based Assays

#### Root Length (RL)

**FIGURE 2:**
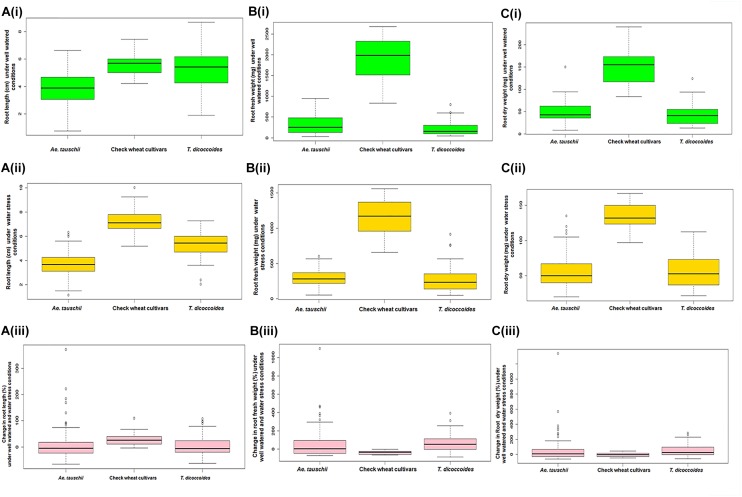
Boxplot representation of root traits of accessions of *Ae. tauschii*, *T. dicoccoides* and check wheat cultivars under well watered and water stress conditions. **(A)** Root length under **(Ai)** well watered, **(Aii)** water stress conditions, and **(Aiii)** change in root length under well watered and water stress conditions. **(B)** Root fresh weight under **(Bi)** well watered, **(Bii)** water stress conditions, and **(Biii**) change in root fresh weight under well watered and water stress conditions. **(C)** Root dry weight under **(Ci)** well watered, **(Cii)** water stress conditions, and **(Ciii)** change in root dry weight under well watered and water stress conditions.

**FIGURE 3:**
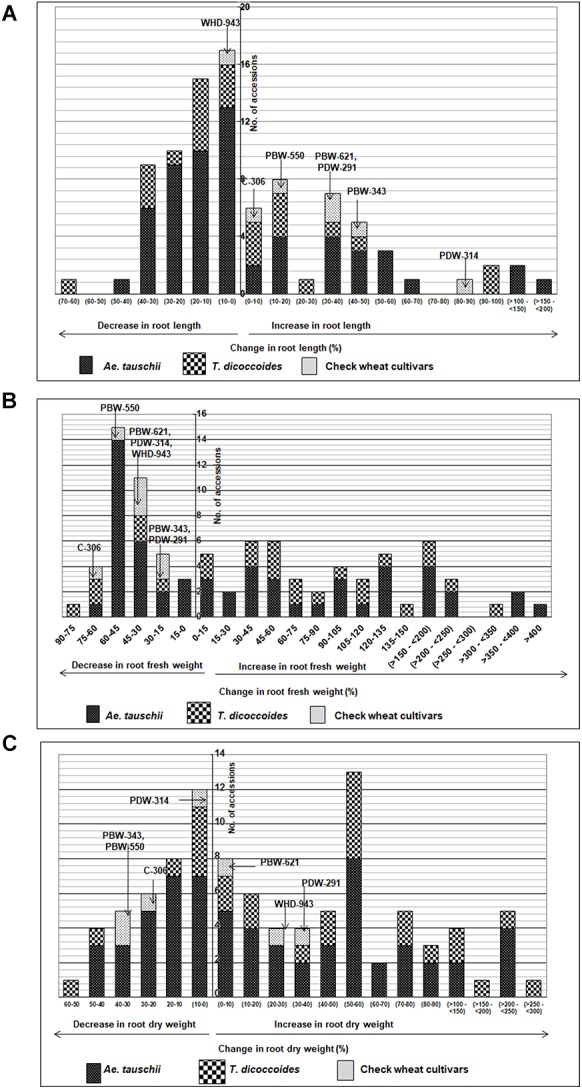
Distribution of accessions of *Ae. tauschii*, *T. dicoccoides* and check wheat cultivars with regard to change in **(A)** root length, **(B)** root fresh weight, and **(C)** root dry weight under well watered and water stress conditions.

Across both well-watered and water-stress regimes, root length tends to increase with an increase in ploidy level, i.e., from diploid (*Ae. tauschii*) to tetraploid (*T. dicoccoides*) accessions and further on to hexaploid wheats (Figure 2Ai,ii). Under well-watered conditions, large number of accessions across the groups clustered in the length range of 4–6 cm when measured on 14 days old seedlings. Average root length for both wild species decreased under water stress (3.82 cm to 3.66 cm for *Ae. tauschii* and from 5.40 to 5.26 cm for *T. dicoccoides*) (Table 1 and Figure 2Ai,ii), yet several accessions showed root elongation (Figure 2Aiii). *Ae. tauschii* accessions 9803, 9814, 14191, 14128, 14226, 14109 and *T. dicoccoides* accessions 5364 and 7130 presented greater than 50% increase in root length under water stress (Table 3). Diversity was wider for the increase than the decrease in root length (Figure 3A). Under water stress conditions, maximum reduction in root length (60%) was seen in a *T. dicoccoides* accession (7120, Supplementary Table S5), whereas highest water stress-mediated induction (197.06%) was observed in an *Ae. tauschii* accession (9803) (Table 3 and Supplementary Table S5). In case of *T. dicoccoides*, the maximum increase in root length (97.47% in accession 5364) was higher than the greatest increase for this trait observed in cultivated set (85% in PDW-314) (Figure 3A).

**Table 3:** Genotypes showing high levels of plasticity for different morpho-physiological traits across well watered and water stress conditions.

Traits	*Ae. tauschii*	*T. dicoccoides*	Cultivars
Root length (cm)	14122^∗^ (36.82%), 14119^∗^ (46.32%), 14109 (53.42%), 14226^∗^ (55.72%), 14128 (57.85%), 14191 (101.50%), 9814 (112.54%), 9803 (197.06%)	4667 (63.43%), 7130 (91.53%), 5364^∗^ (97.47%)	PDW-314^∗^ (85%)
Root fresh weight (mg)	9816 (364.42%), 14109^∗^ (374.85%), 14128 (483.33%)	7056^∗^ (148.66%), 4657 (163.01%), 5364^∗^ (198.22%), 5259 (215.25%), 4667 (311.36%)	–
Root dry weight (mg)	9809 (200%), 9810^∗^ (206.67%), 9816 (246%), 9814^∗^ (250.40%), 14109^∗^ (294.75%)	7079^∗^ (46.02%), 7108^∗^ (75.58%), 13992 (167%), 7130 (205%), 14004 (254%)	PDW-291 (34%)
Shoot length (cm)	14191 (20.20%)	7130 (29.55%),	PDW-291 (0.96%)
Shoot fresh weight (mg)	14191 (18.6%)	–	–
Shoot dry weight (mg)	9809 (43.75%), 9803 (94.74%)	7108^∗^ (14.66%), 7056^∗^ (47.70%), 7054 (59.18%), 7079^∗^ (64.89%), 7130 (246.67%)	–
Proline content (mg g^-1^ FW)	14122 (202.21%), 14128 (205.53%), 14096 (230.66%), 14170^∗^ (337.79%), 14113^∗^ (366.61%), 14169^∗^ (398.31%), 14173^∗^ (418.09%), 14119^∗^ (430.70%)	7130 (211.76%), 5259^∗^ (255.07%), 4654 (329.84%), 4655 (468.06%), 5364 (477.19%), 4656 (835.14%)	C-306^∗^ (606.25%)
Membrane injury (%)^#^	14178^∗^ (21.43%), 9799^∗^ (24.66%), 14240^∗^ (29.30%), 3491^∗^ (30.32%), 3769^∗^ (34.73%), 3761 (37.31%), 14170 (37.33%)	7079^∗^ (43.46%), 4655^∗^ (49.49%), 14004-2^∗^ (54.13%)	C-306^∗^ (58.27%)

*^#^Recorded only under water stress conditions. ^∗^Combines high plasticity and high absolute value (present in top non-significant group with respect to absolute values under water stress conditions, please see details in Supplementary Table S5).*

#### Root Fresh Weight (RFW)

The spectrum of variation for root biomass (fresh and dry weight) distribution under well-watered and water stress conditions was much broader than that observed for root length. Wide genotypic variability could be identified for root fresh weight both within and between the three groups of germplasm (Figure 2B). Under both well watered and water stress conditions, demarcation between cultivated wheats and wild species was quite distinct as check wheat cultivars (both bread wheat and durum wheats) developed very high root biomass (1110–2587 mg) relative to both the species of its wild progenitors. With regard to wild accessions, *Ae. tauschii* dominated the upper limits of root biomass development (50–890 mg) under well watered conditions (Figure 2Bi). However, under PEG-induced decreased water availability, *T. dicoccoides* accessions occupied the higher range of 49–841 mg (Figure 2Bii).

Although cultivated wheats (as a group) developed better average fresh root biomass than the wild species under both well watered and water stress regimes (Figure 2Bi,ii), an overall reduction (15–75%) in root fresh weight (RFW) was noticed in them (Figure 2Biii). No cultivated wheat showed an increase in root fresh weight under water stress. Wild species, on the other hand, demonstrated wide variation with respect to this developmental plasticity. Thirty one *Ae. tauschii* accessions and 19 *T. dicoccoides* accessions showed increase in fresh root biomass (ranging from 3 to 483%) under water stress (Figure 3B), the highest value for *Ae. tauschii* and *T. dicoccoides* being recorded by accession 14128 and 4667, respectively (Table 3).

#### Root Dry Weight (RDW)

As with root fresh weight, bread wheat and durum wheat cultivars maintained greater root dry mass under both well watered and water stress conditions. Whereas the upper limit for root dry matter accumulation was 123 mg for wild accessions (Table 1 and Figure 2Ci), it was almost double for the cultivated set (222 mg). Better part of the wild accessions (nearly 80% of both *Ae. tauschii* and *T. dicoccoides*) accumulated root dry biomass in the range of 25–75 mg under both well watered and water stress conditions (Figure 2Ci,ii). Relative to the changes observed in root fresh weight where data for check wheat cultivars revealed an overall reduction under water stress (Figure 2Ciii), root dry matter exhibited a mixed trend toward induced and/or retarded dry matter accumulation (Figure 3C). Within the wild germplasm pool, the range of positive changes in root dry matter accumulation was wider (up to 300%) than that in which decrease in root dry mass was observed (up to 60%) (Figure 3C). Some of the cultivated wheats registered an increase in root dry weight under stress, but the percentage increase (34%) was lower than that observed in wild wheats (295%) (Figure 3C). Among the 25 evaluated accessions of *T. dicoccoides*, only seven witnessed curtailment in root dry matter under water stress. Rest 18 experienced a net increase in root dry weight. Within *Ae. tauschii* group, accessions were more or less uniformly distributed in the respective lots (25 experiencing net reduction and 33 net gain in root dry weight under stress). Greater than 200% increase in root dry weight was found in *Ae. tauschii* accessions 9809, 9810, 9814, 9816, 14109 and *T. dicoccoides* accession 7130, 13992, and 14004 (Table 3). For the germplasm set used in the present study, water stress seemed to induce a wide range of re-partitioning of resources allocated to different plant parts. The pattern varied markedly for cultivated and wild wheats.

#### Shoot Length (SL)

**FIGURE 4:**
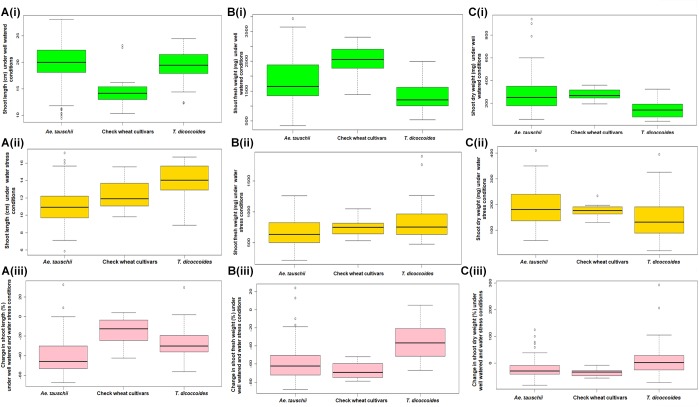
Boxplot representation of shoot traits of accessions of *Ae. tauschii*, *T. dicoccoides* and check wheat cultivars under well watered and water stress conditions. **(A)** Shoot length under **(Ai)** well watered, **(Aii)** water stress, and **(Aiii)** change in shoot length under well watered and water stress conditions. **(B)** Shoot fresh weight under **(Bi)** well watered, **(Bii)** water stress, and **(Biii)** change in shoot fresh weight under well watered and water stress conditions. **(C)** Shoot dry weight under **(Ci)** well watered, **(Cii)** water stress, and **(Ciii)** change in shoot dry weight under well watered and water stress conditions.

**FIGURE 5:**
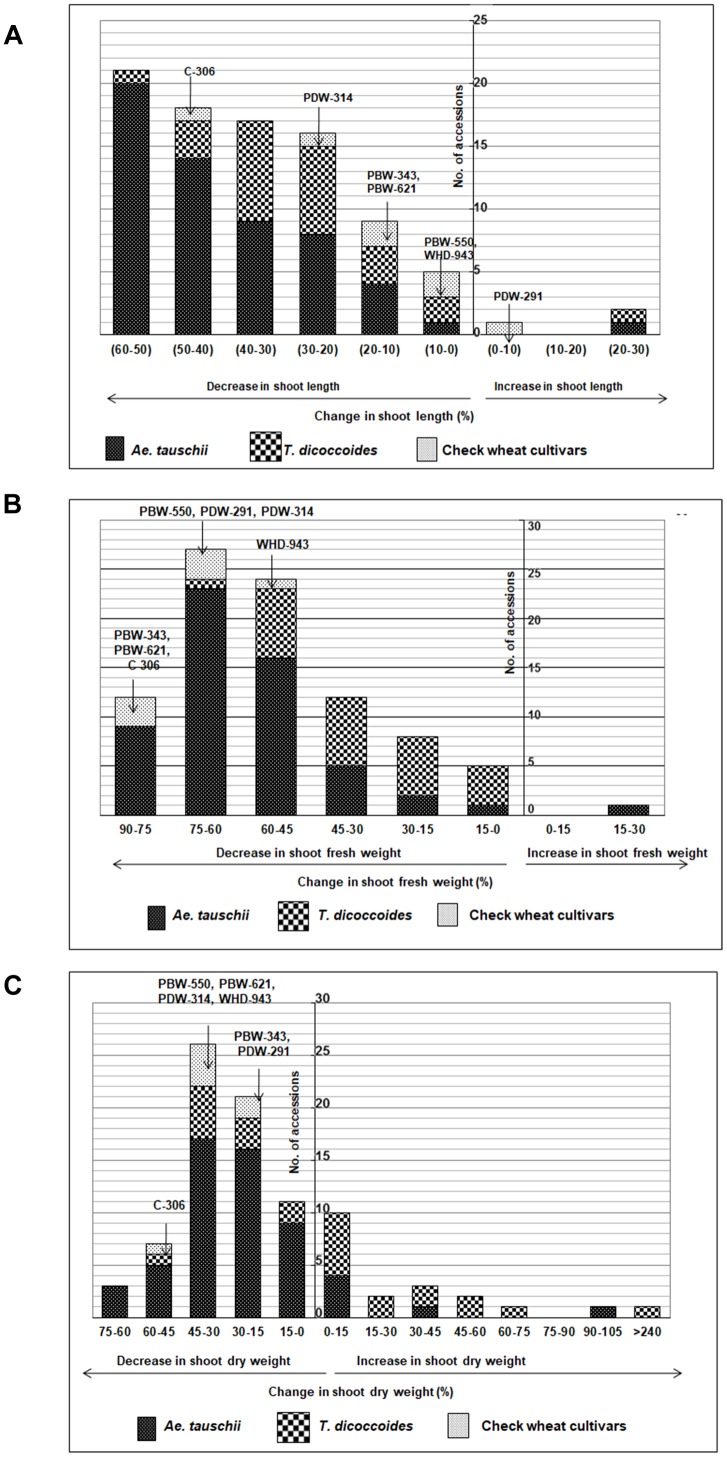
Distribution of accessions of *Ae. tauschii*, *T. dicoccoides* and check wheat cultivars with regard to change in **(A)** shoot length, **(B)** shoot fresh weight, and **(C)** shoot dry weight under well watered and water stress conditions.

Within the present germplasm set, wider distribution range for shoot length was observed in the well watered (Figure 4Ai) than the water stressed set (Figure 4Aii). In well watered set, shoot length varied from 9.9 to 27.72 cm in *Ae. tauschii* and 12.35–23.57 cm for *T. dicoccoides* (Table 1 and Figure 4Ai). However, in water stress set, shoot length varied from 7.17 to 16.75 cm in *Ae. tauschii* and 9.23 to 16.43 cm for *T. dicoccoides* (Table 1 and Figure 4Aii). Where cultivated wheats exhibited root elongation under water stress, shoot length, by and large was suppressed in this group (Figure 4Aii,iii). Low water potential developed due to PEG treatment resulted in up to 60% reduction in shoot length (Figure 5A). Under water stress, a substantial increase in shoot length of the order of 20.20% (*Ae. tauschii* 14191) and 29.55% (*T. dicoccoides* 7130) was observed in progenitor species (Figure 5A). These accessions had also shown a 101.50 and 91.53% increase in root length, respectively (Table 3).

#### Shoot Fresh Weight (SFW)

Among the wilds, *Ae. tauschii* accessions acquired very high shoot fresh weight under well watered conditions that ranged between 460 and 3790 mg (Figure 4Bi). In water stressed set, this upper limit of shoot biomass, however, came down for all the three groups of germplasm (Figure 4Bii), to almost 50% of that observed under well-watered conditions (3790 mg). Maximum number of accessions lay in the range of 500–750 mg shoot biomass within the water stress set (Figure 4Bii). Where *Ae. tauschii* and check wheat cultivars exhibited maximum shoot fresh weight up to 1250 mg, a *T. dicoccoides* accession stood as an outlier as it developed shoot fresh weight of 1837 mg under water stress. Bread wheat and durum wheat cultivars experienced 45–90% reduction in shoot fresh weight (Figure 4Biii). Interestingly, 25 wild accessions showed less than 45% fresh weight reduction under water stress (Figure 5B). An *Ae. tauschii* accession 14191 was the only accession in the present set to have exhibited an increase in shoot fresh weight (18.6%). It had also shown an increase in shoot and root length under water stress.

#### Shoot Dry Weight (SDW)

*Ae. tauschii* exhibited greatest variability in shoot dry matter accumulation within well watered set, evident from the extremely high value of shoot dry weight of a few outlier *Ae. tauschii* accessions (Figure 4Ci). Within the water stressed set, majority of the germplasm lines had shoot dry weight in the range of 100–200 mg (Figure 4Cii). Several wild accessions (*Ae. tauschii* and *T. dicoccoides*) accumulated shoot dry matter more than the maximum shoot dry matter accumulated by wheat cultivars under water stress. Further, though an overall reduction in shoot dry matter was evident, abundant genotypic variation existed among the wild species with respect to change in shoot dry weight under stress (Figure 4Ciii). An average reduction in shoot dry weight within the cultivated pool spanned a range of 15–60% (Figure 5C). Wide genotypic variation existed between wild species accessions with respect to change in shoot dry weight under water stress. An increase in shoot dry weight was observed in two accessions of *Ae. tauschii* 9803, 9809 and in 14 of the 25 accessions of *T. dicoccoides* (up to 240%) (Figure 5C and Table 3). These and other genotypes where stress induced increase in root length or biomass helped minimize reduction in (even if not improve) shoot growth represent true adaptive plasticity.

#### Proline Accumulation in Leaf Tissues

**FIGURE 6:**
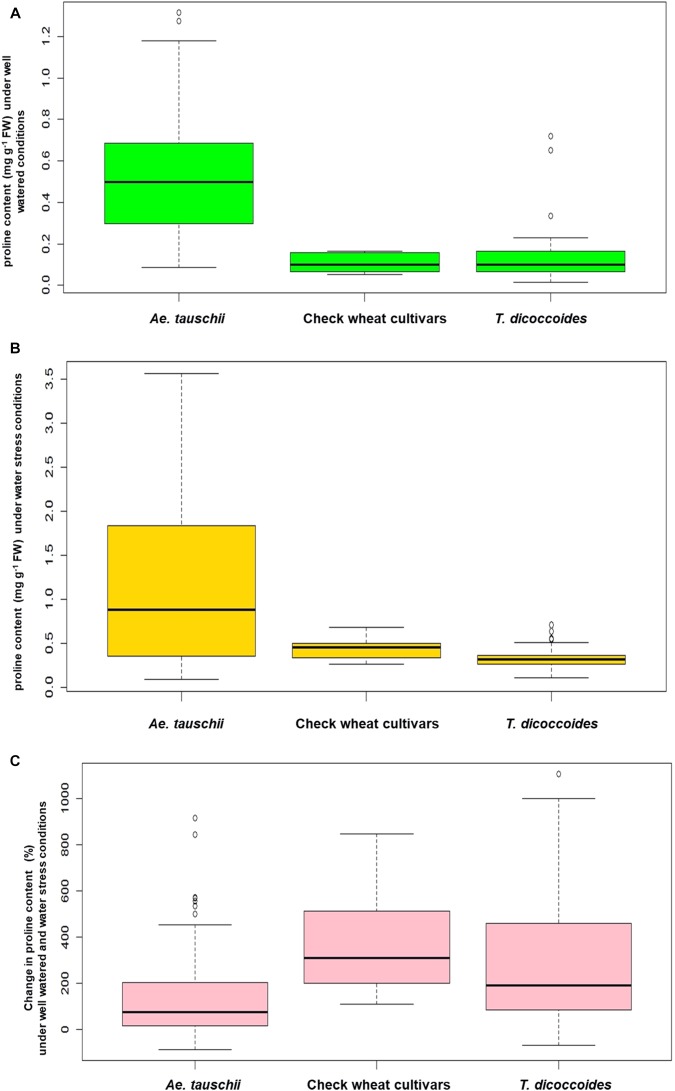
Boxplot representation of proline content in accessions of *Ae. tauschii*, *T. dicoccoides* and check wheat cultivars **(A)** under well watered, **(B)** water stress and **(C)** change in proline content under well watered and water stress conditions.

Inter- and intra-specific variation in the extent of proline accumulation, a well known water stress responsive metabolite was analyzed under field conditions. Under well-watered conditions, all the cultivated wheats and majority of the *T. dicoccoides* accessions maintained a relatively lower basal levels of proline (less than 0.25 mg g^-1^ FW) in their leaf tissue (Figure 6A). *Ae*. *tauschii* accessions seem to go far beyond this range, as is evident from their higher values of proline accumulation that varied from 0.25 to 1.25 mg g^-1^ FW under well watered conditions (Table 2). Effect of differential water regimes was quite dramatic as an increase in proline content under water stress conditions emerged as a common feature across all the three groups of germplasm (Figure 6B). The mean proline content in the *T. dicoccoides* rose from 0.13 to 0.33 mg g^-1^ FW under water stress (Table 2). The average value of proline accumulated by *Ae. tauschii* accessions under water stress conditions (1.12 mg g^-1^ FW) almost doubled as compared to its content under well-watered conditions (0.5 mg g^-1^ FW). More than 50% of the *Ae. tauschii* accessions accumulated amount of proline more than the highest levels of proline accumulated by *T. dicoccoides* and check wheat cultivars under stress (Figure 6B).

With respect to degree of proline induction under water stress, wide genotypic variability could be identified both within and between the three species of wheat (Figure 6C). C-306, a rain-fed bread wheat cultivar of pre-green revolution era demonstrated a 606.25% rise in proline content under stress (Table 3). Higher levels of proline inducibility were observed in the accessions of *T. dicoccoides* that varied from 50 to 850% (Figure 6C). The highest level of proline induction was found in *T. dicoccoides* accession 4656 which exhibited an increase of 835.14% under water stress (Supplementary Table S6). While both constitutive (un-induced) and elevated (induced) levels of proline were higher in *Ae. tauschii*, comparatively lower degree of proline induction (up to 450%) was observed in them. Nine of the 26 *T. dicoccoides* accessions and seven of the 57 *Ae. tauschii* accessions registered greater than 250% increase in proline content under water stress (Supplementary Table S6).

#### Membrane Injury in Leaf Cells

**FIGURE 7:**
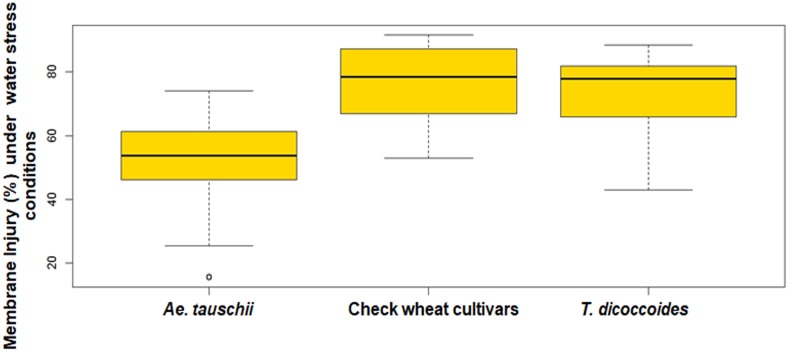
Boxplots representation of per cent membrane injury in accessions of *Ae. tauschii*, *T. dicoccoides* and check wheat cultivars under water stress conditions.

The screening of field grown wild accessions for membrane injury using PEG-6000 revealed ample genetic variability both within and between the three groups of germplasm- *Ae. tauschii*, *T. dicoccoides*, and cultivated wheats. Per cent membrane injury ranged from 21.43 to 89.38% (Table 2). Among cultivated wheats, drought adapted variety C-306 showed minimum membrane injury of 58.27% (Table 3) as against WHD-943, which suffered a damage of 89.38% (Supplementary Table S6). Among the accessions of *Ae. tauschii*, membrane injury levels ranged from 21.43 to 66.86% (Figure 7). *Ae. tauschii* accessions 9799, 14178, 14240 experienced membrane damage between 20 and 30% (Table 3), thus qualifying as accessions maintaining maximum cell membrane stability under water stress. Eight additional lines revealed membrane injury up to 40%. It could be seen that within a set of 57 *Ae. tauschii* accessions, 20 suffered membrane damage lower than the minimum injury seen in a check wheat cultivar, i.e., C-306 (58.27%). As could be seen from the boxplot representation (Figure 7), the lower membrane injury spectrum was primarily occupied by *Ae. tauschii*, whereas higher injury range was populated by *T. dicoccoides* and checks. Compared to *Ae. tauschii*, *T. dicoccoides* displayed relatively higher membrane injury levels, as all accessions lay in the range of 43.46–87.19%. *T. dicoccoides* suffered an average membrane damage of 73% (Table 2), which was higher than the maximum membrane injury noted in *Ae. tauschii* (66.86% in 14211). Nevertheless, membrane injury levels lower than C-306, i.e., 49.49% (accession 4655) and 43.46% (accession 7079) could be identified in *T. dicoccoides*. Later analysis revealed that higher cell membrane injury may be desirable for inducing stress adaptive plasticity in other morpho-physiological traits.

### Change in Agronomic and Productivity Related Traits Due to Water Stress: Use as Tolerance Indices

Plasticity assays presented above were confined to easily quantifiable, vegetative stage traits. The possibility of early-stage stress adaptive plasticity translating into improved stress tolerance needed to be probed. In other words, if a genotype records less or no reduction under water stress in a seedling assay (i.e., high adaptive plasticity) do we expect greater resilience or lesser reduction in an advanced stage productivity related trait? Four prospective tolerance indices based on changes in trait value for days to flowering (tolerance index 1), plant height (tolerance index 2), spike length (tolerance index 3), and grain weight (tolerance index 4) have been employed in the present study (Supplementary Table S7). Variation in these traits is presented and subsequently used to determine correlation with stress adaptive plasticity for seedling and leaf tissue based traits.

**FIGURE 8:**
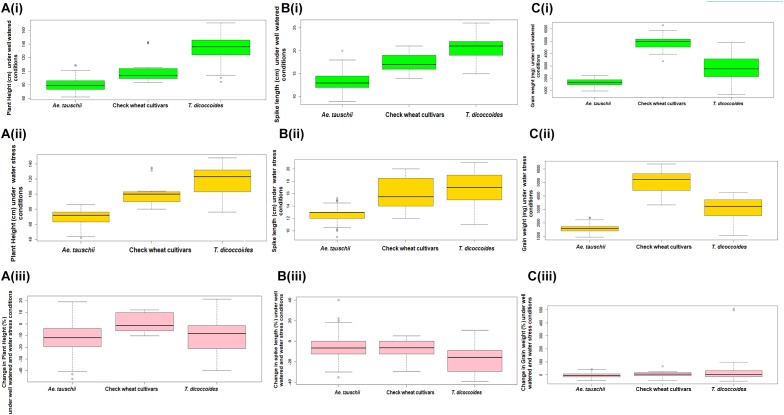
Boxplots representation of phenotypic traits of accessions of *Ae. tauschii*, *T. dicoccoides* and check wheat cultivars under well watered and water stress conditions. **(A)** Plant height under **(Ai)** well watered, **(Aii)** water stress, and **(Aiii)** change in plant height under well watered and water stress conditions. **(B)** Spike length under **(Bi)** well watered, **(Bii)** water stress, and **(Biii)** change in spike length under well watered and water stress conditions. **(C)** Grain weight under **(Ci)** well watered, **(Cii)** water stress, and **(Ciii)** change in grain weight under well watered and water stress conditions.

Analysis of variance carried out for phenotypic traits evaluated over two crop seasons 2010–11 and 2011–12 on the present wheat germplasm set revealed significant effect of genotype, water stress regime, year and their respective interactions (Table 4). BLUP values for these traits were estimated across the years and used to conduct further analysis in the form of correlations and hierarchical clustering. Different accessions of *Ae. tauschii* took 107–127 days to flower under well watered conditions, which reduced to a period between 99 and 124 days under water stress conditions. *T. dicoccoides* accessions, on the other hand, took 105–124 days for heading under well-watered and 103–122 days to flower under water stress conditions. Cultivated wheats flowered earlier, i.e., between 88 and 109 days under well watered and 85–104 days under water stress conditions. Plant height, in case of *Aegilops tauschii*, ranged from 65.00 to 106.00 cm under well-watered and 44.33–85.00 cm under water stress conditions (Figure 8Ai–iii). Several *Ae. tauschii* accessions suffered minimum decline and maintained their plant height even under water stress conditions. Compared to *Ae. tauschii* and check wheat cultivars used, the present set of *T*. *dicoccoides* seemed to encompass an upward shifted spectrum of genetic variation for plant height- 89.33–170 cm under well watered and 76.33–147.33 cm under water stress conditions (Figure 8Ai,ii). *Triticum dicoccoides* faced an overall reduction in average plant height under water stress conditions (average height of 128.20 and 115.45 cm, respectively, in well watered and water stress set), i.e., maximum 41% reduction in plant height was observed in wild species as compared to 8% reduction observed in check wheat cultivars (Figure 8Aiii).

**Table 4:** Genotypic variation in wheat germplasm set for field based traits recorded under well watered and water stress conditions.

Traits/Sources of variation	df	Mean square values			
		Days to flowering (DTF)	Plant height (PLT)	Spike length (SPK)	Grain weight (GW)
Genotype (G)	88	497.85^∗^	6859.76^∗^	90.28^∗^	288076.65^∗^
Water stress regime (W)	1	3270.75^∗^	29847.46^∗^	654.3^∗^	43056.58^∗^
Year (Y)	1	530.91^∗^	64569.69^∗^	2553.26^∗^	670422.48^∗^
G X W	88	48.61^∗^	488.08^∗^	11.61^∗^	22011.78^∗^
G X Y	88	32.12^∗^	91.74^∗^	2.51^∗^	1884.63^∗^

*^∗^Significant at 0.05 level.*

With respect to spike length, range varied from 10 to 18 cm in *Ae. tauschii* and 15.6–25 cm in *T. dicoccoides* under well watered conditions (Figure 8Bi). The spike length range shifted to 9.6–14.8 cm in *Ae. tauschii* and 11.33–21 cm in *T. dicoccoides* under water stress conditions (Figure 8Bii). As far as grain weight is concerned, with increase in ploidy levels, grain weight was found to increase for three groups of species under both water regimes (Figure 8C). The grain weight varied from 1140 to 2145 mg (well watered) to 1155–2248 mg (water stress) for diploid progenitor *Ae. tauschii.* In case of tetraploid *T. dicoccoides*, this range lay between 1134 and 4315 mg (well watered) to 1785–4248 mg (water stress). The variation in present day check wheat cultivars extended beyond this limit as they developed heavier grains to the extent of 3925–6068 mg under well-watered and 4413–5805 mg under water stress conditions. However, with regard to change in grain weight under water stress, an increase in grain weight was observed in several accessions (Figure 8Ciii).

### Associations Within and Between Early Stage Adaptive Plasticity and Tolerance Indices

Correlations between length and biomass observations recorded on the same morphological trait (e.g., root length) were observed as per expectation. However, remarkably strong positive correlations emerged between root and shoot trait based plasticities (Table 5). For instance, positive associations could be seen between changes in root and shoot length (*r* = 0.447, *n* = 87), change in root length and change in shoot dry weight (*r* = 0.470). Change in root dry weight revealed slight good correspondence with both change in shoot fresh weight (*r* = 0.251) as well as change in shoot dry weight (*r* = 0.369). This was unexpected with respect to balanced growth hypothesis where root adaptive responses to stress are often at cost of shoot growth. These positive correlations indicated that root responses were truly adaptive in nature. In genotypes which responded to water stress by increasing root growth, the reduction in shoot length was less severe, compared to genotypes which could not respond by an increase in their root growth.

**Table 5:** Correlations between changes in trait values used for the study of stress adaptive plasticity and BLUP values based tolerance indices under water stress conditions.

	ΔProline	ΔRL	ΔRFW	ΔRDW	ΔSL	ΔSFW	ΔSDW	ΔDTF	ΔPLT	ΔSPK	ΔGW
MI	0.227^∗^	0.135	0.149	0.123	0.332^∗∗^	0.296^∗∗^	0.276^∗∗^	0.372^∗∗^	0.093	0.004	0.359^∗∗^
ΔProline		0.108	0.053	0.03	0.033	-0.058	0.007	0.065	0.225^∗^	0.016	-0.06
ΔRL			0.351^∗∗^	0.440^∗∗^	0.447^∗∗^	0.301^∗∗^	0.470^∗∗^	0.286^∗∗^	0.089	0.006	0.134
ΔRFW				0.546^∗∗^	0.09	0.172	0.101	-0.008	-0.006	0.214^∗^	0.02
ΔRDW					0.187	0.251^∗^	0.369^∗∗^	0.202	0.09	0.092	0.267^∗^
ΔSL						0.586^∗∗^	0.478^∗∗^	0.352^∗∗^	-0.057	-0.07	0.404^∗∗^
ΔSFW							0.603^∗∗^	0.174	-0.074	-0.11	0.299^∗∗^
ΔSDW								0.268^∗^	0.057	-0.058	0.526^∗∗^
ΔDTF									0.053	-0.023	0.279^∗∗^
ΔPLT										0.219^∗^	-0.1
ΔSPK											-0.202

*^∗^Correlation significant at the 0.05 level; ^∗∗^Correlation significant at the 0.01 level. Δ Change under water stress. MI, Membrane injury; RL, root length; RFW, root fresh weight; RDW, root dry weight; SL, shoot length; SFW, shoot fresh weight; SDW, shoot dry weight; DTF, days to flowering; PLT, plant height; SPK, spike length; GW, grain weight.*

Membrane injury seems to work as an excellent stress adaptive plasticity induction mechanism as indicated by significant positive association with all the three shoot characters i.e., change in shoot length (*r* = 0.332), change in shoot fresh weight (*r* = 0.296) and change in shoot dry weight (*r* = 0.276). Growth induction for shoot based characters reflected their better stress adaptive plasticity of shoots than roots. Further, membrane injury was positively correlated with change in the content of proline (*r* = 0.227) and change in grain weight under water stress (*r* = 0.359). Genotypes showing higher membrane injury under water stress suffered lower reduction in root and shoot parameters as well as grain weight. This may not be plausible if we regard membrane injury as a stress induced damage, but can be explained to some extent if membrane injury serves as a stress signal for activating adaptive responses. Out of the four tolerance indices, tolerance index based on plant height and spike length correlated weakly with plasticity indices (Table 5). Tolerance based on days to flowering correlated well with several plasticity indices (*r* = 0.372 with membrane injury, *r* = 0.286 with change in root length, *r* = 0.352 with change in shoot length, *r* = 0.268 with change in shoot dry weight). Strongest impact of early stage plasticity indices was, however, observed on grain weight based tolerance index which was positively associated with change in root dry weight under water stress (*r* = 0.267), change in shoot fresh weight (*r* = 0.299), change in shoot length (*r* = 0.404) and change in shoot dry weight (*r* = 0.526). These correlations furnish a link between field and seedling based plasticities.

**FIGURE 9:**
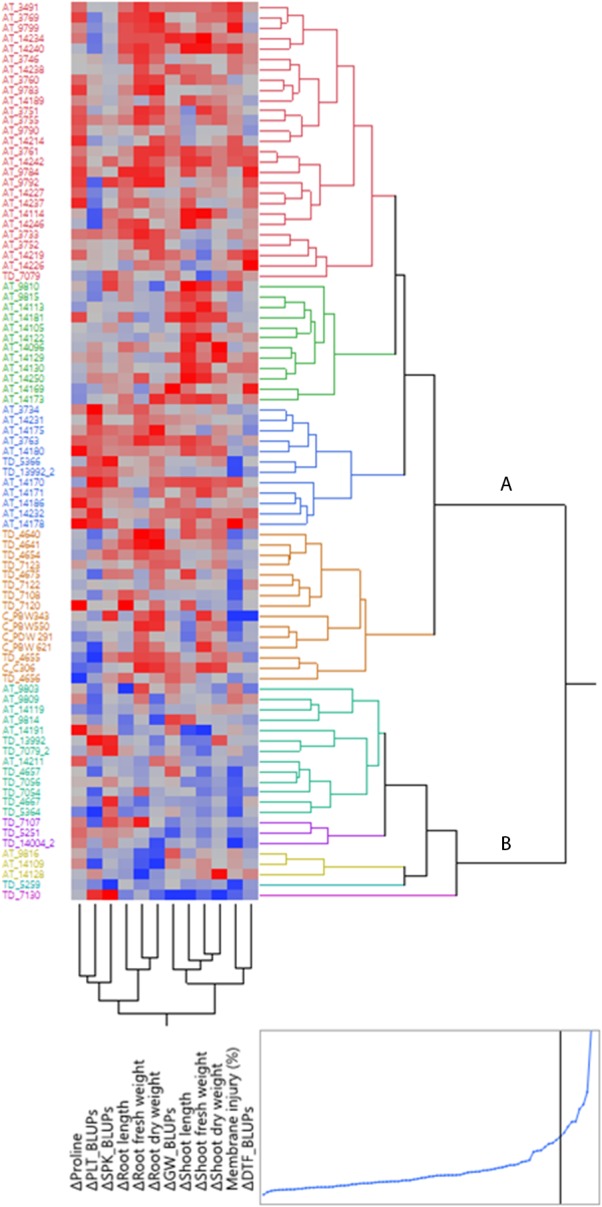
Hierarchical cluster analysis (HCA) of early stage adaptive plasticity traits and BLUP derived tolerance indices in wheat germplasm set. AT, TD, and C correspond to accessions of *Aegilops tauschii*, *Triticum dicoccoides* and check wheat cultivars, respectively. Each accession is visualized in a single row and each trait value is represented by a single column. Red indicates unfavorable change or reduction in trait value, whereas less of the unfavorable change or increase in trait value is depicted in blue.

Based on Best linear unbiased predictor (BLUP) values of plasticity scores and tolerance indices, HCA, employing Ward’s Method was performed using squared euclidean distance matrix to elucidate genotypic variation within the present germplasm for responsiveness to water stress. The overall stress adaptive response of three groups of species with respect to various water stress plastic traits is presented as a heat map (Figure 9). The heat map is based on change in trait values observed across well watered and water-stress conditions. Stress adaptive plasticity seemed to have a strong genotypic basis rather than an exclusive property of a species or a group. Nevertheless, strong species based trends were also visible. The genotypic clustering formed two major groups- the smaller group (cluster B) represented by resilient genotypes which showed either less of the unfavorable change or in some cases positive change under water stress conditions. This group consisted of nine *Ae. tauschii* and 13 *T. dicoccoides* accessions. Together, these constitute the group of genotypes possessing greater stress adaptive plasticity. Among these, three *Ae. tauschii* accessions 9816, 14109, 14128 and two *T. dicoccoides* accessions 5259 and 7130 emerged as the five most stress adaptive genotypes considering all the traits. Out of these, accessions 14109 and 7130 have been marked for showing higher stress adaptive plasticity for root elongation and dry matter accumulation and accession 5259 for greater root fresh weight acquisition and higher proline induction under water stress conditions. These progenitor accessions have been used in wheat breeding programme at our center to develop synthetic wheats. Cultivated wheats, on the heat map, were placed in the larger group (cluster A) representing moderate to low stress adaptive behavior. Notably, the cultivated types formed a close cluster and represented moderate levels of plasticity. Apparently, the wild species had a larger spectrum of variation and some of them constituted the group representing the least adaptive behavior.

## Discussion

Wild progenitors have been widely used as donors of resistance to biotic stresses such as powdery mildew (Rong et al., 2000), yellow rust (Gill and Raupp, 1987; Goodman et al., 1987; Cox et al., 1990), and karnal bunt (Villareal et al., 1995). Similarly, genes for productivity traits from *Ae. tauschii* (Gororo et al., 2002) and for higher grain weight and protein content from *T. turgidum* var *dicoccoides* have also been transferred to *T. aestivum* (Kushnir and Halloran, 1984; Mesfin et al., 2000). Our center has successfully used marker based strategy to tag and transfer several disease resistance genes (e.g., *Lr57*/*Yr40*, *Lr76*/*Yr70*, *Lr58*) from wild species to the wheat breeding pipeline. The wild progenitors have been used less extensively as donors of abiotic stress tolerance though *T. dicoccoides* (Peleg et al., 2005) and *Ae. tauschii* (Kurahashi et al., 2009) have been targeted for drought (Peleg et al., 2005), high temperature (Pradhan et al., 2012), or salinity stress (Siasho et al., 2016). Trait plasticity as a component of productivity and stress tolerance remains a future goal, even though wild species are known to grow and thrive in alternatively harsh and favorable conditions.

The set of wild accessions on which this study is based have also been a part of other reports in literature. For instance, Shah et al. (2000) found *Ae. tauschii* accessions 3733 and 3734 to be totally immune to leaf rust. A new source of greenbug resistance has been derived from *Ae. tauschii* accession 9783 (Weng et al., 2005). Likewise, *T. dicoccoides* accessions 4657 and 4675 exhibited an intermediate fusarium head blight (FHB) reaction, whereas accession 5259 was found susceptible to FHB reaction (Oliver et al., 2007). Besides these reports, entire set of lines used in the study has also been characterized for various traits of economic interest at our center (Chhuneja et al., 2010, 2015; Gupta et al., 2010; Suneja, 2014; Suneja et al., 2015a,b, 2017; Arora et al., 2017). A direct hybridization approach to gene transfer from *Ae. tauschii* Coss. to *Triticum aestivum* has also been developed (Seghal et al., 2011). This cross referencing of *Ae. tauschii* and *T. dicoccoides* accessions to other studies indicates all round worth of the lines and is likely to promote their judicious use in future wheat breeding programmes.

With accessions of diploid (*Ae. tauschii*) and tetraploid (*T. dicoccoides*) progenitor species as the core genetic material, the present study was aimed to decipher how plasticity indices based on seedling and vegetative traits are able to correlate with agronomic and productivity related tolerance indices. Between these two sets of traits, there are several developmental steps which are likely to dimnish the influence of early stage stress adaptive behavior. There is a possibility that these simple early stage stress adaptive plasticity indices may represent a broader based plasticity mechanisms operating in these genotypes. The strategy of using changes in trait values as we shift from non-stress to stress conditions rather than *per se* trait values under stress allowed a delineation of stress adaptive plasticity. The association of early stage and more likely to be adaptive responses with later stage productivity based tolerance indices emerged as an important finding. Having established a strong genetic basis for the plasticity phenomenon, in this (by design) broad spectrum germplasm set, the identification of donors open up several follow up avenues.

### Genotypic Variation for Stress Adaptive Plasticity in Root-Shoot Traits

In this study, marked induction behavior in terms of root development (increase in root length and dry weight) came to light in some of the wild species accessions. Kadam et al. (2015) found root length of wheat increased in response to water deficit stress and reported plasticity in root length, thickness, root weight density, xylem diameter and vessel number along the length of the root. An increase in root: shoot ratio and absolute root mass in response to moisture stress has been previously reported in wheat by Blum et al. (1983) and Reynolds et al. (2007). Balanced growth hypothesis (Bloom et al., 1985) suggests that some plants respond to drought by stimulating or maintaining root growth while reducing shoot growth. Using Lockhart’s equation, Hsiao and Xu (2000) elucidated that the underlying mechanism behind shift in allometry are the differences in the sensitivity of root and shoot growth to water stress.

At the level of stress administered in the present study, the responses went beyond redistribution of resources between root and shoot to stress adaptive plasticity as revealed by positive correlation between root and shoot based plasticity. This resulted from genotypes which responded to water stress by increasing root growth and consequently maintained better shoot length, compared to genotypes which could not respond by an increase in their root growth. While this phenomenon has been mentioned in results, genotypes going even one step further in their responses need to be mentioned. Greater adaptation to water stress than well-watered conditions was observed in *Ae. tauschii* accession 14191 and *T. dicoccoides* accession 7130, where in addition to increase in the relative size of root (adaptive plasticity of 101.50 and 91.53%, respectively), shoot growth displayed a stress adaptive plasticity of 20.20 and 29.55%, respectively. Similarly, *Ae. tauschii* accession 9803 expressed stress adaptive plasticity of the order of 94.74% in shoot dry weight and 34% in root dry weight. These lines proved to be notable exceptions to the norm as an increase in both root- and shoot length was evident under water stress. Greater adaptation to water stress could be found in some wild accessions where root length/weight is relatively low under well watered conditions. These accessions seem to have greater adaptation to water stress than well watered conditions. Such remarkable responses warrant a strong genetic basis.

### Proline: High Inducibility but Complex Role

Lower basal levels of proline in *T. dicoccoides* (in comparison to *Ae. tauschii*) under well watered conditions may hint toward their local adaptation to arid climates of Israel and regions of North Crescent where *T. dicoccoides* originated. Abundant genetic variation in water stress induced proline accumulation was identified in accessions of *Arabidopsis thaliana* (Verslues and Juenger, 2011; Kesari et al., 2012; Verslues et al., 2014). Accessions from generally drier regions have lower proline accumulation. Accessions that habitually face drought may have other metabolic adjustments such that higher levels of proline may not be needed as long as a particular threshold level of osmotic potential is maintained in the cell (Kesari et al., 2012). *T. dicoccoides* evolved in a relatively restricted geographic region, i.e., eastern Mediterranean region, characterized by a long, hot dry summer and a short, mild wet winter with fluctuating amounts and distribution of rainfall (Loss and Siddique, 1994; Peleg et al., 2005). Stress responsive higher proline induction in *T. dicoccoides* may be aligned to dynamic up-regulation of *P5CS1* (proline biosynthesis) or down-regulation of *ProDH* (proline degradation). *Ae. tauschii* that showed higher constitutive but lower levels of proline induction, is known to be adapted to a more continental climate of Central Asia. However, further investigations are necessary to reveal the background of high proline content in *Ae. tauschii* accessions. AABB-genome through its metabolic plasticity and DD-genome through heightened basal expression together might have contributed in enhancing the fitness of natural hexaploid wheat across diverse eco-geographical environments. A similar observation has also been reported in a study where the expression pattern of *HKT1;5* was studied in 2x (diploid), 4x (tetraploid), nat-6x (natural hexaploid) and neo-6x (synthetic hexaploid) genomes of wheat in response to salt stress (Yang et al., 2014).

### Inter-Trait Associations: Uncovering Network of Plasticities

Three remarkable observations with respect to network of plasticities emerged and may serve as lead for further studies. First is the positive correlation between root and shoot based stress adaptive plasticities which deviated from the generally observed resource allocation to roots at the cost of shoots under water stress (Hsiao and Xu, 2000; Weiner, 2004; Gargallo-Garriga et al., 2014). Second is concerning membrane injury serving as a signal or trigger for stress adaptive plasticity and thus showing positive association with various morpho-physiological attributes. Third important observation related to the marked inducibility in proline accumulation in leaf tissue, but its largely negative connotations for stress adaptive plasticity. Finally, the association of early stage and more likely to be adaptive responses with later stage productivity based tolerance indices emerged as an important finding. The associations observed in this study point toward the larger perspective that wild species are able to capitalize on plasticity to ensure fitness in variable environments (Vilela and Gonzalez-Paleo, 2015).

### Opportunities for Genetic Analysis and Molecular Marker Tagging

Identifying genes responsible for drought response has been challenging because of polygenic nature as well as issues concerning easy evaluation of these abiotic stress responsive traits. Inducible traits would be even harder to pursue in breeding programmes, but molecular marker assisted selection may prove to be a powerful tool as demonstrated by the success in transfer of submergence tolerance gene in rice (Bailey-Serres et al., 2010). Root growth angle as a trait was not targeted in the present study, however, this trait is receiving increased attention due to cloning of *DRO1* (Deep Rooting) locus in rice (Uga et al., 2013). Recent reviews foresee optimization of root system architecture (RSA) as the basis of second green revolution (Meister et al., 2014). Attempts to identify a gene or a set of genes that control the switch for shift in root-shoot allometry under water stress are at present largely lacking. Genetic and molecular marker analysis for induction of root growth under water stress at the diploid level using contrasting *Ae. tauschii* parents (accessions 9803, 9814, 14109, and 3769) offers itself as a feasible prospect. Inducible traits represent the best option in the face of expected variations in stress over space and time.

### Identification of Potential Donors

On the whole, many wild accessions could be identified as suitable donors for a suite of water stress responsive traits. *Aegilops tauschii* accessions 9816 and 14109 revealed higher stress adaptive plasticity in terms of increased root biomass (fresh and dry weight) under stress. *Aegilops tauschii* accession 14128 exhibited root elongation and higher proline induction under water stress. *Aegilops tauschii* accession 9809 increased root and shoot dry weight under stress. This accession also displayed physiological plastic responses in terms of increased activity of ROS scavenging enzymes under drought stress (Suneja et al., 2017). Within *T. dicoccoides* group, accession 7130 displayed root and shoot elongation, increased root and shoot dry matter accumulation and higher proline induction under stress. *T. dicoccoides* accession 5259 too accumulated more proline and acquired higher root fresh weight under conditions of decreased water availability. These genotypes represent a situation where well watered condition seems to be more stressful than the water stress (at the level of stress administered in this study). This accession-specific behavior invites opportunities for molecular genetic analysis of inducibility under stress as a trait, associated pleiotropic effects, if any, for eventual introgression into elite wheat cultivars. Crosses have been conducted between *T. dicoccoides* accession 5259, 7130 and *Ae. tauschii* accessions 9816, 14109, and 14128 to develop synthetic hexaploid wheats that might combine favorable drought responsive traits from AABB- and DD-genome of wild progenitors of wheat, leading to enhanced trait expression due to gene interaction. Subsequent crosses with high yielding wheat cultivars may help tailor their genetic makeup that enables them to thrive and perform well under conditions of unanticipated or variable environmental stress. Ideally, a winning combination of root and shoot traits along with appropriate metabolic switches may be successfully met to enhance water stress resilience of present day wheat cultivars.

## Author Contributions

YS conducted the experiments, generated data, carried out the analysis, and prepared the draft of the manuscript. AG and NB conceived the idea, designed and supervised the study, interpreted results, revised, and finalized the manuscript.

## Conflict of Interest Statement

The authors declare that the research was conducted in the absence of any commercial or financial relationships that could be construed as a potential conflict of interest.
